# Supplementary material for: How Can We Best Measure Frailty in Cardiosurgical Patients?
Source: J Clin Med. 2023 Apr 20;12(8):3010. doi: 10.3390/jcm12083010 (PMC10140958; doi:10.3390/jcm12083010)
Supplement: Supplementary file 1 [file jcm-12-03010-s001.zip › jcm-2263790-supplementary.pdf]

Table S1: Comorbidities and Type of Surgery in 3 groups according to fried.

Comorbidities	All (246)	Frail (16)	Prefrail (130)	Control (100)	p frail vs. prefrail	p frail vs. control	p prefrail vs. control
Age (years)	66.5+/-9.05	67.12+/-7.61	66.62+/-9.54	66.23+/-8.67	1	1	1
Sex (female)	21.14% (52)	43.75% (7)	23.08% (30)	15% (15)	0.273	0.052	0.273
Hypertension	72.36% (178)	81.25% (13)	70.77% (92)	73% (73)	1	1	1
Diabetes	30.49% (75)	31.25% (5)	35.38% (46)	24% (24)	0.259	1	1
LVEF	56.86+/-8.3	58.12+/-4.9	56.45+/-9.31	57.18+/-7.29	1	1	1
Euroscore II	2.59+/-3.28	5.71+/-8.41	2.6+/-2.84	2.08+/-1.91	0.223	<0.001	<0.001
BMI (kg/m2)	29.71+/-5.21	31.44+/-6.79	29.77+/-5.41	29.37+/-4.64	0.564	0.419	0.451
Surgery					0.281	0.03	0.281
CABG	40.24% (99)	25% (4)	37.69% (49)	46% (46)			
sAVR	32.11% (79)	31.25% (5)	31.54% (41)	33% (33)			
Combination CAGB/sAVR	13.41% (33)	6.25% (1)	13.85% (18)	14% (14)			
Other Operation	14.23% (35)	37.5% (6)	16.92% (22)	7% (7)			
Complications							
In hospital mortality	4.88% (12)	6.25% (1)	4.62% (6)	5% (5)	1	1	1
1-year-mortality	8.22% (18)	26.67% (4)	7.02% (8)	6.67% (6)	1	0.14	0.14
Wound healing disorder	11.48% (28)	13.33% (2)	11.63% (15)	11% (11)	1	1	1
Stroke during stay	3.28% (8)	0% (0)	3.88% (5)	3% (3)	1	1	1
Arrythmias	25.61% (63)	26.67% (4)	26.36% (34)	25% (25)	1	1	1
Re-Thoracotomy	5.69% (14)	6.67% (1)	5.47% (7)	6% (6)	1	1	1
Myocardial infarction	0.82% (2)	6.67% (1)	0% (0)	1% (1)	1	1	0.58
Pneumonia	5.33% (13)	13.33% (2)	3.88% (5)	6% (6)	1	1	0.985
Delirium	10.25% (25)	13.33% (2)	11.63% (15)	8% (8)	1	1	1
